# Use of real-world data as pivotal evidence in veterinary regulatory applications

**DOI:** 10.3389/fvets.2025.1588068

**Published:** 2025-06-18

**Authors:** Raffaele Bruno

**Affiliations:** Zoetis Veterinary Medicine Research & Development, Zaventem, Belgium

**Keywords:** veterinary, medicines, regulatory affairs, real-world data, real-world evidence

## Abstract

Real-world data (RWD) has the potential to complement or serve as an alternative to randomized clinical trials (RCTs) in veterinary medicine, mirroring trends observed in human medicine. Sourced from diverse platforms including digital databases and wearable devices, RWD may provide valuable insights into the effectiveness, safety, and broader societal impacts of veterinary medicinal products. Although its role as pivotal evidence in veterinary drug submissions remains limited due to challenges related to data quality, methodological rigor, and regulatory acceptance, reflections on its potential applications in the veterinary domain are already possible.

Drawing parallels to its role in human medicine development, real-world data (RWD) holds significant promise for future applications in veterinary medicine, potentially to complement or even partially replace standard randomized clinical trial (RCTs) data in regulatory submissions.

Although a common global regulatory definition of RWD is not yet available (1), both the FDA and EMA converge on the definition of RWD as patient data derived from a variety of sources such as electronic health records from clinics or laboratories, patient registries, prescription data, and information generated from wearables or collected via apps in a home setting (2, 3). Additional veterinary specific RWD sources include the data generated by animal owners, remote health sensing devices, data from robotic milking systems, or from slaughterhouses (4). These sources often involve digital technologies for data generation, collection, or availability; however, the association with digital technologies is not a prerequisite for classification as RWD. The European definition more precisely categorizes RWD based on routine data collection from sources other than traditional clinical trials (3), while the FDA definition does not exclude data from clinical trials. The real-world evidence (RWE) is the information derived from the analysis of one or more RWD sources and which can become pivotal evidence of effectiveness or safety once included within regulatory applications.

The post-authorization collection of information through pharmacovigilance systems is a well-established example of RWE providing critical safety data for both human and veterinary medicinal products, often becoming pivotal evidence to support label amendments of authorized therapies. However, the acceptance of RWD for generating evidence of clinical effectiveness and supporting regulatory approval of innovative products or new therapeutic indications remains limited. In human medicine, RWD/RWE contributed to innovation directly influencing the regulatory decision by serving as external or historical comparators for single-arm trials, comparing surrogate and clinical endpoints, and assessing the effectiveness between treatment groups; in other cases, the RWE role was more limited, providing supportive information to the standard datasets such as incidence, prevalence, or evolution of diseases (5–7). The EMA has recently qualified a primary endpoint based on data passively collected by digital and wearable devices in home settings, paving the way for new methods of evaluating treatment effectiveness in real-life conditions, particularly for indications impacting ambulatory function (8).

While examples in the veterinary field are more limited, regulatory authorities have previously accepted applications based on data generated under real-life conditions that, according to current classification criteria, could be defined as RWD/RWE. For instance, the bibliographic applications, where applicants may substitute original safety and efficacy studies with published scientific literature, represent a registration route supported by data collected from a variety of sources other than standard clinical trials, consistent with the definition of RWD (9–11); however, this option has always been limited to well-established active substances with an already recognized level of effectiveness and safety. Another example is the effectiveness of new medicinal products assessed for regulatory purposes via wearable devices in the home setting, but as part of standard clinical controlled studies (12, 13).

The growing interest in RWD is driven by advancements in digital technologies, which provide novel solutions for data generation at the individual and patient level, as well as for the storage and accessibility of large datasets. This interest extends to companies developing veterinary medicines, which are eager to leverage these emerging data sources, similar to their counterparts in human medicine. However, the challenges and limitations associated with accepting RWE as pivotal evidence in regulatory submissions for human medicines are equally relevant in the veterinary sector. These challenges include issues related to data quality and heterogeneity, variations in data collection practices across regions, the design and methodology of data collection, the statistical analysis plan underpinning data interpretation and the potential for bias and measurement errors (3, 14–16). Given the unique characteristics of the animal health sector, some of these challenges are likely to be more pronounced in the veterinary field, where the typical technology-driven sources of RWD—such as electronic health records, e-health services, insurance claims and billing data—are less widely adopted for animal patients. For example, the SAVSNET network—which integrates a centralized database of anonymized electronic health records from UK veterinary practices and diagnostic laboratories (17)—has no comparable system in other EU Member States. Addressing concerns about RWD relevance and reliability, the representativeness of the fewer available databases and the generalizability of the information obtained will be critical (4). Furthermore, the approval of alternative data collection methods may necessitate rigorous review before being approved for use (8) and such demanding qualification processes could lead to prohibitive costs within the veterinary sector.

However, despite the aforementioned challenges, RWD may offer several potential advantages for innovation in veterinary medicine, some of which are specific to the veterinary sector:

- **Long-term Evaluation of Safety and Effectiveness:** As the medicalization of companion animals increases, particularly in the management of chronic conditions associated with an aging pet population, the need to assess the long-term safety and effectiveness of new veterinary products becomes increasingly important. RWE offers novel and potentially cost-effective alternatives to standard clinical trials for such long-term assessments. To address the limited availability and adoption of electronic health records in the veterinary field, data could be generated directly by pet owners as observer-reported-outcomes (ObsRO) (18) and reported via commonly used digital devices (19) like for patient-reported outcomes (PROs) (20) in human medicine. Following an initial assessment by a veterinarian, the acceptance of ObsRO—whether as the standalone source or complementary to clinical data—will likely depend on the extent to which a parallel interpretation by a clinician is required. Nevertheless, owner reported outcomes could significantly reduce the costs associated with prolonged clinical monitoring while enabling the capture of response to therapies in a home setting. In addition, the regulatory acceptance of long-term validation of new product performances through RWE could facilitate the registration process of therapies supported by limited datasets and that necessitate the generation of additional evidence post-registration (21, 22).
- **External controls:** For clinical trials in humans, regulatory authorities have accepted external controls derived from historical data or real-world settings instead of traditional randomized controls in situations when the use of an internal control poses ethical or feasibility challenges (23, 24). This approach has been applied to severe conditions, such as oncology, where withholding life-saving treatments is unethical, or rare diseases, where recruiting sufficient participants is impractical. Such reasons are equally applicable to veterinary trials investigating efficacy in life-threatening conditions lacking positive controls or rare indications. But external controls could also address ethical concerns specific to veterinary studies. For instance, the use of a negative control in assessing the preventative efficacy against parasitic infections or transmission of vector-borne diseases may raise ethical concerns in the absence of adequate rescue protocols for infected animals or show low number of infections in the negative group due to the unpredictability of actual exposure (25, 26). Conversely, a positively controlled study for these indications might yield results of limited value if both treatment groups demonstrate 100% preventative efficacy, as this provides no insight into the underlying infection pressure. In such cases, epidemiological data and records from veterinary clinics and diagnostic labs could serve as RWD to establish external controls in single-arm trials, offering insights into theoretical infection rates among untreated animals in specific trial areas. Despite challenges and potential biases in external historical controls (27), regulatory authorities increasingly accept alternative evidence when traditional RCTs are impractical or insufficient (14).
- **Data from wearable devices:** The growing adoption of wearable devices in veterinary medicine offers novel avenues for the continuous and passive collection of RWD on animal health and behaviors. Devices like activity-tracking collars for companion animals can evaluate treatment effects on conditions that affect ambulatory function such as pain, inflammation, cardiac issues, or behaviors like scratching. This approach could also indirectly inform assessments of health-related quality of life (HRQoL). Wearable accelerometers have been employed to quantify scratching episodes associated with chronic dermatologic conditions in dogs (28, 29). Data from these devices have also been included in marketing authorization applications for products for the treatment of chronic musculo-skeletal disorder in cats (12) and anxiety in dogs (13). In livestock, biosensors, either detachable or imprinted, can monitor body temperature, feeding patterns and parameters related to milking performance in dairy cows (30). These developments provide opportunities for veterinary-specific uses of RWD, not only on the individual animal patient health status but also on production parameters, a key factor in the livestock industry. The integration of digital health technologies in veterinary medicine has the potential to enhance objectivity in clinical assessments. Digital measurements from wearable devices reduce reliance on subjective evaluations by veterinarians or owners, minimizing variability caused by human interpretation. This technological advancement parallels trends seen in human healthcare, where the European Medicines Agency (EMA) recently qualified the 95th percentile of stride velocity (SV95C) as a primary endpoint in the evaluation of Duchenne Muscular Dystrophy (DMD) therapies, with data collected passively using wearable devices (8) as alternative to the standard 6-min walking test (6MWT). The SV95C allows continuous, real-world monitoring in home settings, reducing bias from timing, patient motivation or other subjective factors. The same principles apply to veterinary medicine, where digital tools can reduce reliance on subjective assessments, which in the veterinary field are further complicated when reported by owners rather than by clinicians. In addition, the continuous data collection in real-world settings would alleviate the stress-induced bias animals may exhibit during veterinary examinations, allowing for a more accurate reflection of their everyday health status. While the data quality challenges associated with mobile device usage in regulatory decisions for human health (16) are equally pertinent to veterinary medicine, the benefits of objective, continuous, and passive measurements emphasize the transformative potential of wearable technology in enhancing regulatory evaluations of veterinary treatments.
- **Measure effects beyond the individual animal patient:** Unlike human medicine, veterinary medicine assessments extend beyond individual patients to broader societal impacts, such as antimicrobial resistance, human food safety, and environmental implications. These non-target animal “safeties” must be addressed both in the marketing authorization application dossier and during post-registration phase. Thanks to advancements in digital technologies and regulations in these areas, rapidly accumulating RWD is becoming available for these aspects. For example, veterinary electronic prescriptions (31) combined with large country-specific databases (32) can reveal trends in antimicrobial use. New vaccines or immunomodulators aimed at disease prevention may reduce antimicrobial use, and these reductions could be quantified and potentially reflected on product labeling (33). Regarding food safety, data on veterinary medicinal product residues in animal-derived food products is now publicly available, with results provided per active substance, country, and target species (34). Additionally, environmental impacts, such as methane emissions, can be measured at the individual animal level (35). Real-World Evidence (RWE) derived from these RWD sources is more likely to support new label claims for existing products than the approval of new ones, as sufficient data emerges only after extensive market use. However, such data can also indirectly support the registration of new veterinary medicinal products. For instance, RWD can be used to feed post-authorization risk management or monitoring plans included in the initial marketing authorization, giving regulators added assurance that safety aspects will continue to be evaluated post-registration in real-world conditions.

In conclusion, the use of real-world data (RWD) in veterinary regulatory applications holds great potential to complement or, in certain cases, replace traditional randomized clinical trials (RCTs). While challenges related to data quality, collection methodologies, and region-specific practices remain, the growing availability of digital technologies offers new ways to harness data from diverse sources. Real-world evidence (RWE) derived from these data can support regulatory decisions, particularly for post-authorization monitoring and the addition of new claims to existing products. As the veterinary sector evolves, leveraging RWD could provide a more comprehensive understanding of product safety and effectiveness under actual usage conditions, while also addressing broader societal impacts such as antimicrobial resistance and environmental sustainability.

## Data Availability

The original contributions presented in the study are included in the article/supplementary material, further inquiries can be directed to the corresponding author.
